# Bmi-1 Promotes Glioma Angiogenesis by Activating NF-κB Signaling

**DOI:** 10.1371/journal.pone.0055527

**Published:** 2013-01-31

**Authors:** Lili Jiang, Libing Song, Jueheng Wu, Yi Yang, Xun Zhu, Bo Hu, Shi-Yuan Cheng, Mengfeng Li

**Affiliations:** 1 Department of Pathophysiology, Guangzhou Medical University, Guangzhou, Guangdong, China; 2 Department of Experimental Research, Cancer Center, Sun Yat-sen University, Guangzhou, Guangdong, China; 3 Department of Microbiology, Zhongshan School of Medicine, Sun Yat-sen University, Guangzhou, Guangdong, China; 4 Department of Pharmacology, Zhongshan School of Medicine, Sun Yat-sen University, Guangzhou, Guangdong, China; 5 Key Laboratory of Tropical Disease Control (Sun Yat-sen University), Chinese Ministry of Education, Guangzhou, Guangdong, China; 6 Department of Neurology, Northwestern Brain Tumor Institute, Center for Genetic Medicine and The Robert H. Lurie Comprehensive Cancer Center, Northwestern University Feinberg School of Medicine, Chicago, Illinois, United States of America; Complutense University, Spain

## Abstract

Angiogenesis in glioma is associated with the poor prognosis of the disease and closely correlates with the highly invasive phenotype of glioma cells, which represents the most challenging impediment against the currently glioma treatments. Bmi-1, an onco-protein, has been implicated in the progression of various human cancers, including gliomas, whereas its role in glioma angiogenesis remains unclear. Our current study examined the effects of Bmi-1 on glioma angiogenesis in vitro as well as in vivo. We found that overexpression of Bmi-1 enhanced, whereas knockdown of Bmi-1 diminished, the capability of glioma cells to induce tubule formation and migration of endothelial cells and neovascularization in chicken chorioallantoic membrane. *In vivo*, Bmi-1 overexpression and knockdown, respectively, promoted and inhibited angiogenesis in orthotopically transplanted human gliomas. Furthermore, NF-κB activity and VEGF-C expression was induced by Bmi-1 overexpression, whereas Bmi-1 knockdown attenuated NF-κB signaling and decreased VEGF-C expression. Additionally suppression of NF-κB activity using a specific chemical inhibitor abrogated the NF-κB activation and the pro-angiogenic activities of glioma cells. Together, our data suggest that Bmi-1 plays an important role in glioma angiogenesis and therefore could represent a potential target for anti-angiogenic therapy against the disease.

## Introduction

Angiogenesis, the formation of new blood vessels from pre-existing ones, occurs in many physiological and pathological conditions such as development, wound healing and cancer [1]. In the past decades, tumor angiogenesis has become an important field of research in cancer biology and clinical oncology, as neovascularization is required for the growth and metastasis of various types of human cancers including gliomas [2]. Tumor cells produce angiogenesis inducers, represented by vascular epithelial growth factors (VEGF), which play a crucial role in endothelial survival, proliferation and differentiation, as well as new vessel sprouting [3]. Endothelial cells of environmental capillaries activated by tumor cells-released proangiogenic factors are commonly subjected to migration and mitotic division, consequently forming a network of new vessels connected with the existing vasculature [4]. Its essential role in cancer development and progression makes angiogenesis, as well as molecular promoters underlying this important process, a promising target for cancer therapy.

Glioma, the most common type of primary brain tumors, represents one of the most aggressive and lethal human cancer types [5]. The prognosis of gliomas has remained poor, and the cumulative 1-year survival rate is below 30% [6], [7]. Of note, malignant gliomas, such as glioblastoma multiforme (GBM), are characterized by highly aggressive phenotypes, including prominent vascularization underlied by upregulation of pro-angiogenic factors and endothelial proliferation [8]. In this context, the poor patient survival is largely attributable to the high invasiveness and accelerated growth of the tumor [9]. It has been reported that invasion and proliferation of glioma cells are angiogenesis-dependent, and that gliomas are among the cancer types with the highest degree of vascularization, which also represents an independent prognostic factor for the disease [9]–[11]. Increased tumor microvascular density (MVD) has been identified as a hallmark in gliomas with a shorter patient survival [12], [13]. Understanding the molecular mechanisms mediating the development and progression of glioma angiogenesis will help identify diagnostic/prognostic biomarkers as well as therapeutic targets against the deadly condition.

B-cell-specific Moloney murine leukemia virus integration site 1 (Bmi-1) is a member of the polycomb gene family and has been found upregulated in a variety of human cancer types including acute myeloid leukemia and breast, colon, lung, ovarian and nasopharyngeal cancers, suggesting a potential role of Bmi-1 as an oncogene [14]–[19]. Dysregulated expression of Bmi-1 has been found in various biological processes associated with cancer development and progression, including cell proliferation, invasion and repression of apoptosis or senescence [18], [20]–[23]. Previous studies have revealed that Bmi-1 interacts with a number of cancer-related signaling pathways, such as activating NF-κB signaling, suppressing the p16/Rb and/or p19^ARF^/MDM2/p53 tumor suppressive pathways, and inducing the activation of Hedgehog signaling [24]–[27]. It has been reported that Bmi-1 induces epithelial-mesenchymal transition (EMT) in nasopharyngeal carcinoma cells through stabilizing Snail, a transcriptional repressor associated with EMT, via modulation of the PI-3K/Akt/GSK-3β pathway [14]. Furthermore, Bmi-1 suppresses transcription of the tumor suppressor phosphatase and tensin homolog deleted on chromosome ten (PTEN) via directly associating with the PTEN gene locus [14]. Moreover, Bmi-1 suppresses p16^INK4a^ and p14^ARF^, and promotes cell proliferation by inhibiting the p16/Rb and/or p14^ARF^/MDM2/p53 signaling pathways [27]. Our previous study has also demonstrated that Bmi-1 is overexpressed, correlates with poor overall survival of patients with gliomas and plays an important role in growth and survival of glioma tumor cells [22]. Whether Bmi-1 has a biological function in glioma angiogenesis however, remains unclear.

In the present study, we report that Bmi-1 promotes glioma angiogenesis in vitro as well as in vivo. We find that Bmi-1 activates the NF-κB signaling pathway and induces expression of NF-κB target genes, including VEGF-C that plays a pivotal role in tumor vascularization. These results reveal a mechanism by which Bmi-1 enables glioma neovascularization via activation of NF-κB signaling.

## Materials and Methods

### Ethics statement

This study was carried out in strict accordance with the recommendations in the Guide for the Care and Use of Laboratory Animals of the National Institutes of Health. All animal studies were conducted with the approval of the Sun Yat-sen University Institutional Animal Care and Use Committee. All surgery was performed under sodium pentobarbital anesthesia, and all efforts were made to minimize suffering.

### Cells and Treatments

Glioma cell lines LN382T and T98 were purchased from ATCC and grown in Dulbecco's modified Eagle's medium (Invitrogen, Carlsbad, CA, USA) supplemented with 10% fetal bovine serum (HyClone, Logan, UT, USA) and 100 units penicillin-streptomycin at 37°C with 5% CO_2_ atmosphere in a humidified incubator. NF-κB inhibitor JSH-23 compound (EMD, La Jolla, CA, USA), dissolved in dimethyl sulfoxide, was used at 30 µM final concentration to treat the cells (37°C, 5% CO_2_). A VEGFC inhibitor, VEGFR3-Fc (GenWay Biotech, CA, USA) dissolved in sterile water, was used at 100 ng/ml to treat cells (37°C, 5% CO_2_).

### Vectors and Retroviral Infection

pMSCV/Bmi-1 overexpressing human Bmi-1 was constructed as previously described [14]. To silence endogenous Bmi-1 expression, RNA interference (RNAi) oligonucleotide (5′-ATGAAGAGAAGAAGGGATT-3′) was cloned into a retroviral transfer vector pSuper-retro-puro, and retroviral production and infection were performed as described previously [27]. Stable cell lines expressing Bmi-1 or silencing Bmi-1 were selected by treatment with 0.5 µg/ml puromycin for 10 days, beginning from 48 hours after infection.

### Real-time RT-PCR and data analysis

Real-time RT-PCR and data analysis were performed as previously described [28]. Briefly, total RNA was extracted from cells by using the Trizol reagent (Invitrogen, Carlsbad, CA, USA) according to the manufacturer's instruction. Two micrograms of RNA from each sample was used for cDNA synthesis primed with random hexamers. Real-time RT-PCR was performed using the Applied Biosystems 7500 Sequence Detection system. PCR amplification of cDNA was performed with a initial denaturation step at 95°C for 10 minutes, followed by 28 cycles of denaturation at 95°C for 60 seconds, primer annealing at 58°C for 30 seconds, and primer extension at 72°C for 30 seconds, and completed with a final extension at 72°C for 5 minutes. The expression of various genes were defined based on the threshold cycle (Ct), and relative expression levels were calculated as 2^−[(C^_T_^of miR-182)−(C^_T_^of U6)]^ after normalization with reference to expression of housekeeping gene GAPDH. PCR primers were designed by using the Primer Express version 2.0 software (Applied Biosystems, Foster City, CA), and the primer sequences are as follows:

*VEGF-C*-forward: GTGTCCAGTGTAGATGAACTC; *VEGF-C*-reverse: ATCTGTAGACGGACACACATG. *TNFα*- forward: CCAGGCAGTCAGATCATCTTCTC; *TNFα*- reverse: AGCTGGTTATCTCTCAGCTCCAC. *IL-6*- forward: TCTCCACAAGCGCCTTCG; *IL-6*- reverse: CTCAGGGCTGAGATGCCG. *CCND1*- forward: AACTACCTGGACCGCTTCCT; *CCND1*-reverse: CCACTTGAGCTTGTTCACCA. *MYC*- forward: TTCGGGTAGTGGAAAACCAG; *MYC*- reverse: CAGCAGCTCGAATTTCTTCC. *Bcl-xL*- forward: ATTGGTGAGTCGGATCGCAGC; *Bcl-xL*- reverse: AGAGAAGGGGGTGGGAGGGTA. *MMP-9*-forward: ACGACGTCTTCCAGTACCGA; *MMP-9*-reverse: TTGGTCCACCTGGTTCAACT. *GAPDH*- forward: ATTCCACCCATGGCAAATTC; *GAPDH*- reverse: AGAGGCAGGGATGATGTTCTG.

### Western Blotting (WB) analysis

WB analysis was performed according to a standard method as previously described [22]. The blot membrane was probed with a 1∶500-diluted rabbit anti-Bmi-1 antibody (Abcam, Cambrige, MA, USA). The membranes were stripped and re-probed with mouse monoclonal anti-β-actin antibody (1∶1,000; Sigma, Saint Louis, MO, USA) as a loading control.

### HUVEC tubule formation assay

HUVEC tubule formation and migration assay was performed as previously described [29]. Briefly, 200 µl growth-factor-reduced Matrigel (Collaborative Biomedical Products) was added into wells of a 24-well plate and polymerized for 30 min at 37°C. HUVEC (2×10^4^) in 200 µl of conditioned medium were added to each well and incubated at 37°C in 5% CO_2_ for 20 h. Images were captured under bright-fields using a Zeiss Axio Imager A1 microscope with 100× final magnification. The induced tube-like structures under each condition in triplicate were quantified by determining their length.

### Chicken chorioallantoic membrane (CAM) assay

To evaluate the direct effect on angiogenesis, CAM assay was performed at the 8th day of development of fertilized chicken eggs as previously described [30]. A 1-cm diameter window was opened in the shell of each egg with 8-day-old chicken embryo (Yueqin Breeding Co. Ltd, Guangdong, China). The surface of the dermic sheet on the floor of the air sac was removed to expose the CAM. A 0.5-cm diameter filter paper was first placed on top the CAM, and 100 µl conditioned medium harvested from transduced or treated glioma cells was added on the center of the paper. After the window was closed with sterile adhesive tape, the eggs were incubated at 37°C under 80–90% relative humidity for 48 hours. Following fixation with stationary solution (methanol: acetone = 1∶1) for 15 min, the CAM was cut and harvested, and gross photos of each CAM were taken under a digital camera (Panasonic, Osaka, Japan). The effect of conditioned media was evaluated by the number of second- and third-order vessels in comparison with that treated with the medium harvested from the vector-control group. Statistical analysis of second- and third-order vessels was carried out using 2-tailed student's t-test.

### Transwell migration assay

Various cells (1×10^4^ in 50 µl) to be tested were plated on the top side of polycarbonate Transwell filter and incubated at 37°C for 22 hrs, followed by removal of cells inside the upper chamber with cotton swabs. Migrated cells on the lower membrane surface were fixed in 1% paraformaldehyde, stained with hematoxylin, and counted (10 random 200× fields per well). Cell counts were expressed as the mean number of cells per field of view. Three independent experiments were performed and the data are presented as mean ± standard deviation (SD).

### Intracranial brain tumor xenografts and IHC staining

Various cells (5×10^5^ in 5 µl) were stereotactically implanted into individual nude mouse brains. Brain glioma-bearing mice were euthanized 2 weeks post-implantation as previously described [29]. Brains of various mice were removed, cut into 6 µm thickness sections and stained immunohistochemically (IHC) staining using anti-CD31 (Invitrogen, Carlsbad, CA, USA) and an anti-VEGF-C antibodies (Cell signaling, Danvers, MA, USA). The images of IHC stained brain sections were captured using the AxioVision Rel.4.6 computerized image analysis system (Carl Zeiss Co. Ltd., Jena, Germany).

### Luciferase assay

Fifteen thousand cells were seeded in triplicates in 24-well plates and allowed to settle for 24 h. 100 ng of luciferase reporter plasmids or the control-luciferase plasmid, plus 10 ng of pRL-TK renilla plasmid (Promega, Madison, WI, USA), were transfected into glioma cells using the Lipofectamine 2000 reagent (Invitrogen, Carlsbad, CA, USA) according to the manufacturer's recommendation. Luciferase and renilla signals were determined 48 h after transfection using the Dual Luciferase Reporter Assay Kit (Promega, Madison, WI, USA) according to a protocol provided by the manufacturer. Three independent experiments were performed and the data are presented as mean ± SD.

### Enzyme-linked immunosorbent assay (ELISA)

ELISA was performed according to the manufacturer's manual (Keygentec Co., Shanghai, China). Briefly, standard (diluted) and tested samples (Bmi-1 overexpressing and silencing cells, and the vector control cells), including a negative control, are added to appropriate wells of the plate. Incubated at 36°C for 90 min, and wash the unbound samples off the plate with DI water and PBS-Triton. Incubation with specific antibody (anti-VEGFC) performed at 36°C for 60 min. After washing step, add secondary antibody and incubate for 60 min. And then add the substrate and incubate at room temperature for 60 min. Stop the reaction and read plates on an ELISA plate reader. Colorimetric measurement was recorded as OD_450_ readings.

### Statistical analysis

Statistical analyses were performed using the SPSS 11.0 software package. Data are presented as mean ± SEM. P values of 0.05 or less were considered statistically significant.

## Results

### Ectopic expression of Bmi-1 induced angiogenesis in glioma cells in vitro

**Figure 1:**
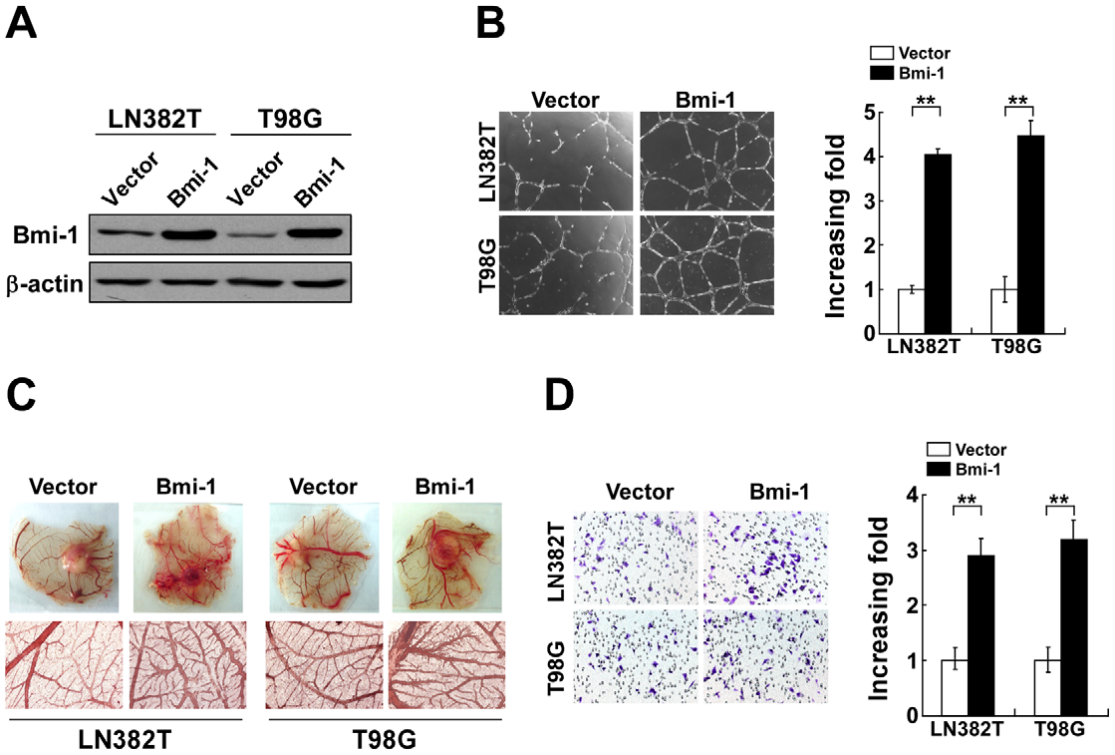
Ectopic expression of Bmi-1 enhances the pro-angiogenic activity of glioma cells *in vitro*. (A) WB analysis of Bmi-1 protein expression in LN382T-vector, LN382T-Bmi-1, T98G-vector and T98G-Bmi-1 cells; β-actin was used as a loading control. (B) Representative images (left) and quantification (right) of HUVEC formed tube-like structures on Matrigel-coated plates with CM derived from indicated cultured cells. (C) Representative images of the CAM blood vessels stimulated by CM derived from indicated cells. The lower panel, ×100 magnification. These experiments were repeated at least three times with similar results. (D) Representative images (left) and quantification (right) of migrated HUVEC cells treated with indicated conditioned medium analyzed in a Transwell migration assay. Vector: pMSCV-vector. Error bars represent the mean ± SD of three independent experiments; ** *P*<0.01.

To investigate the effect of Bmi-1 on glioma angiogenesis, previously constructed pMSCV/Bmi-1-derived retrovirus [22] was used to transduce LN382T and T98G glioma cells to establish Bmi-1-overexpressing stable cell lines. Expression of Bmi-1 was verified by WB (Figure 1A). Subsequently, the effect of Bmi-1 on enhanced ability of glioma cells in inducing in vitro angiogenesis was examined using an *in vitro* tube-like structure formation assay. Conditioned medium (CM) collectd from Bmi-1-overexpressing glioma cells was added to Matrigels embedded with HUVEC cells. As shown in Figure 1B, CM derived from Bmi-1-expressing glioma cells induced more tube-like structure formed by HUVEC as well as in shorter time than that by control CM. Additionally, ectopic expression of Bmi-1 by glioma cells also potently induced the formation of second- and third-order vessels in chicken chorioallantoic membranes (CAM assay) (Figure 1C). Additionally, HUVEC migration was markedly induced by CM of Bmi-1-overexpressing glioma cells compared with control CM (Figure 1D). Taken together, the data suggested that Bmi-1 enhanced the capability of glioma cells to stimulate neovascularization *in vitro*.

### Bmi-1 promoted the expression of VEGF-C in glioma cells in vitro

**Figure 2:**
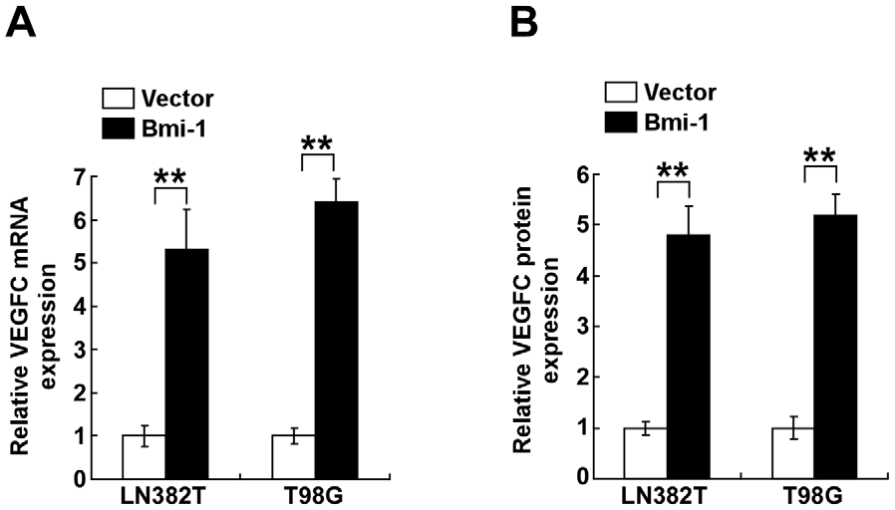
Bmi-1 activates VEGF-C in glioma cells. (A) Real-time PCR quantification of *VEGF-C* mRNA expression in vector-control cells (Vector) and Bmi-1-overexpressing cells (Bmi-1). Levels of mRNA expression are presented as the fold increase relative to that in vector-control cells and normalized to GAPDH. (B) The protein expression of VEGF-C in cell supernatants by ELISA assay. Vector: pMSCV-vector. Error bars represent mean ± SD of three independent experiments; ** *P*<0.01.

It has been reported that new vessel formation driven by glioma cells is mechanically associated with increase expression of VEGF-C [29]. Toward an understanding of the mechanism by which Bmi-1 promotes the angiogenesis of glioma cells, we investigated whether Bmi-1 induces the expression VEGF-C in glioma cells. As shown in Figure 2A, *VEGF-C* mRNA was upregulated in Bmi-1-overexpressing glioma cells when compared that in control cells. In addition, levels of secreted VEGF-C protein in the supernatants of Bmi-1-overexpressing glioma cells was also elevated (Figure 2B). These results indicated that Bmi-1 increased expression and secretion of VEGF-C in glioma cells.

### Silencing Bmi-1 reduced angiogenesis and VEGF-C expression in glioma cells

**Figure 3:**
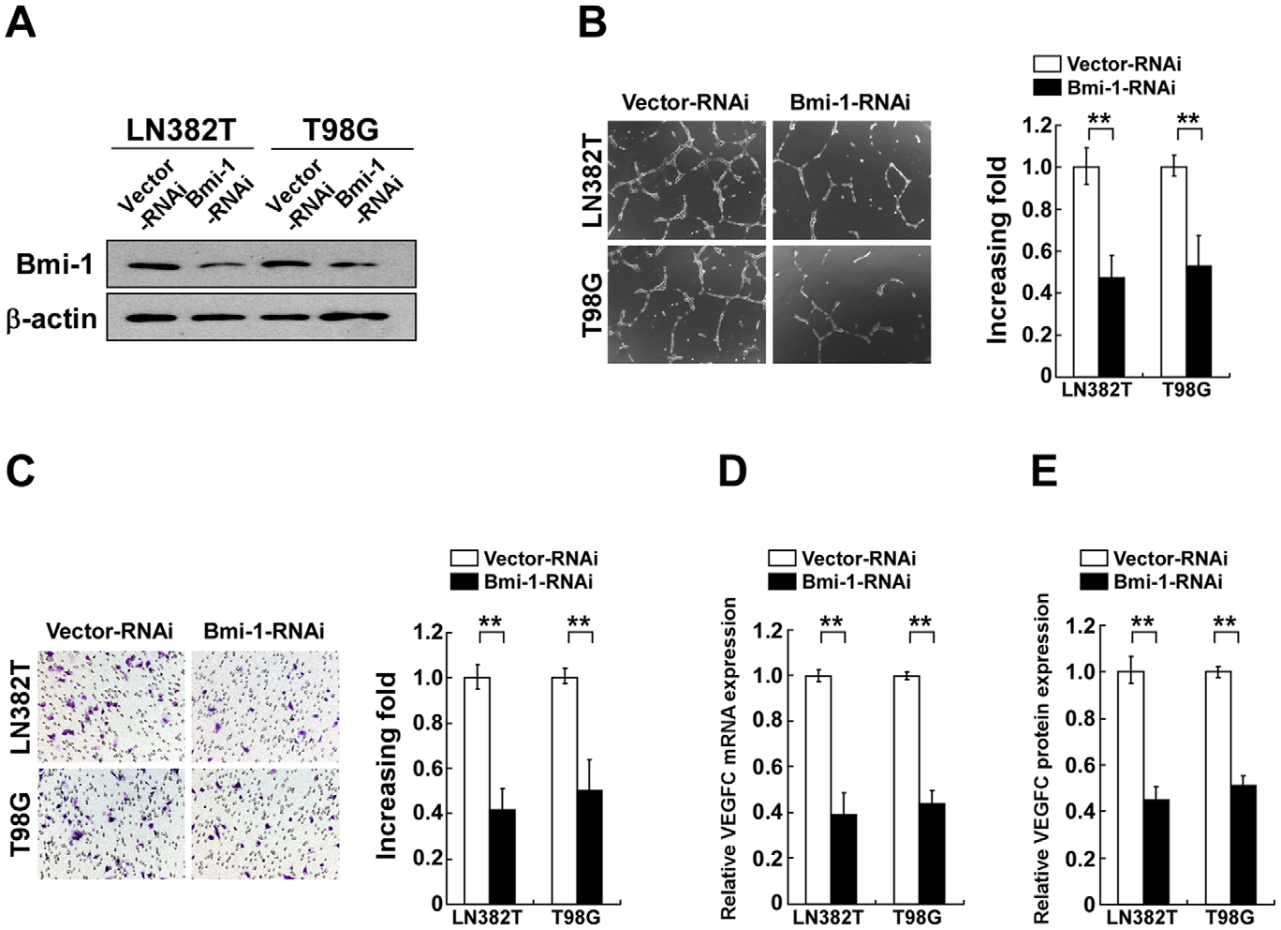
Knockdown of Bmi-1 reduces glioma cell-promtoed angiogenesis in vitro. (A) WB analysis of Bmi-1 protein expression in vector-control cells and Bmi-1-shRNA-transduced glioma cell lines (Bmi-1-RNAi); β-actin was used as a loading control. (B) Representative images (left) and quantification (right) of tube-like structures formed by HUVEC on Matrigel-coated plates with CM derived from indicated cells. The experiments were repeated at least three times with similar results. (C) Representative images (left) and quantification (right) of migrated HUVEC cells treated with indicated CM analyzed in a Transwell migration assay. (D) Real-time PCR quantification of *VEGF-C* mRNA expression levels in vector-control cells and Bmi-1-shRNA-transduced glioma cell lines (Bmi-1-RNAi). Levels of mRNA expression are presented as the fold increase relative to that in vector-control cells and normalized to GAPDH. (E) ELISA assay of VEGF-C protein expression in cell supernatants. Vector-RNAi: pSuper-retro-puro-vector. Error bars represent the mean ± SD of three independent experiments; ** *P*<0.01.

To establish an experimental model in which expression of endogenous Bmi-1 is knocked down, Bmi-1-specific RNA interference (RNAi) oligonucleotides were cloned into a retroviral transfer vector pSuper-retro-puro as described previously [22]. In this model, attenuated expression of Bmi-1 was verified by WB (Figure 3A). Consequently, as shown in Figure 3B, CM of Bmi-1-shRNA-transfected cells showed decreased abilities to induce formation of tube-like structure by HUVEC cells *in vitro* when compared with the control CM, suggesting that inhibition of endogenous Bmi-1 in glioma cells markedly reduced glioma cell-induced *in vivo* angiogenesis. The stimulated HUVEC migration was also reduced by CM derived from Bmi-1-silencing glioma cells when compared with control CM (Figure 3C). Consistent with these observations, shRNA depletion of Bmi-1 in glioma cells also significantly reduced VEGF-C mRNA and protein expression (Figure 3D and E).

### Bmi-1 induced angiogenesis and VEGF-C expression in vivo

**Figure 4:**
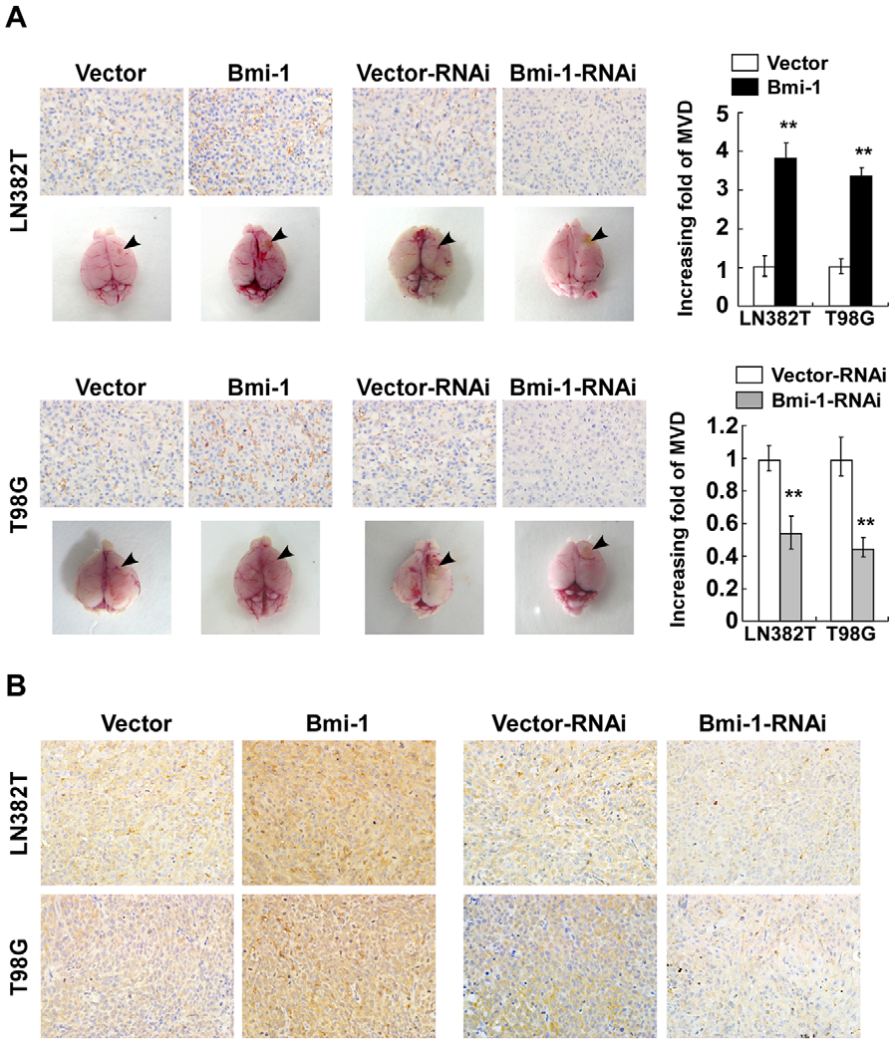
Bmi-1 induces glioma cells angiogenesis *in vivo*. (A) Representative images (left) and quantifications (right) of MVD in indicated brain gliomas by IHC staining using an anti-CD31 antibody. The images below IHC stained tissues are the whole brains of the mice. Arrows, tumor cell inoculation points on the brain. (B) IHC staining of indicated brain gliomas for VEGF-C expression. Vector: pMSCV-vector, Vector-RNAi: pSuper-retro-puro-vector. Error bars represent mean ± SD of three independent experiments; **P<0.01.

To assess whether Bmi-1-induced glioma angiogenesis *in vivo*, we utilized an orthotopic brain glioma tumor model and determined whether Bmi-1 modulates glioma angiogenesis in the brain. As shown in Figure 4A, when compared with the controls, gliomas that stably overexpressed Bmi-1 markedly induced vascellum on the surface in the tumor-bearing brain whereas surface vascellum was significantly decreased in brains bearing Bmi-1-knockdown gliomas. Furthermore, IHC analyses demonstrated markedly elevated expression of CD31, enhanced microvascular outgrowth and increased MVD in Bmi-1-overexpressing gliomas, as opposed to those Bmi-1-knockdown gliomas (*P*<0.05, Figure 4A). Additionally, when compared with control gliomas, Bmi-1 overexpression up-regulated VEGF-C levels whereas knockdown of Bmi-1 resulted in decreased VEGF-C expression in brain gliomas (Figure 4B). Taken together, our data demonstrated that Bmi-1 induced tumor angiogenesis and VEGF-C expression in brain gliomas.

### Bmi-1 promoted the pro-angiogenic potency of glioma cells via the NF-κB pathway

**Figure 5:**
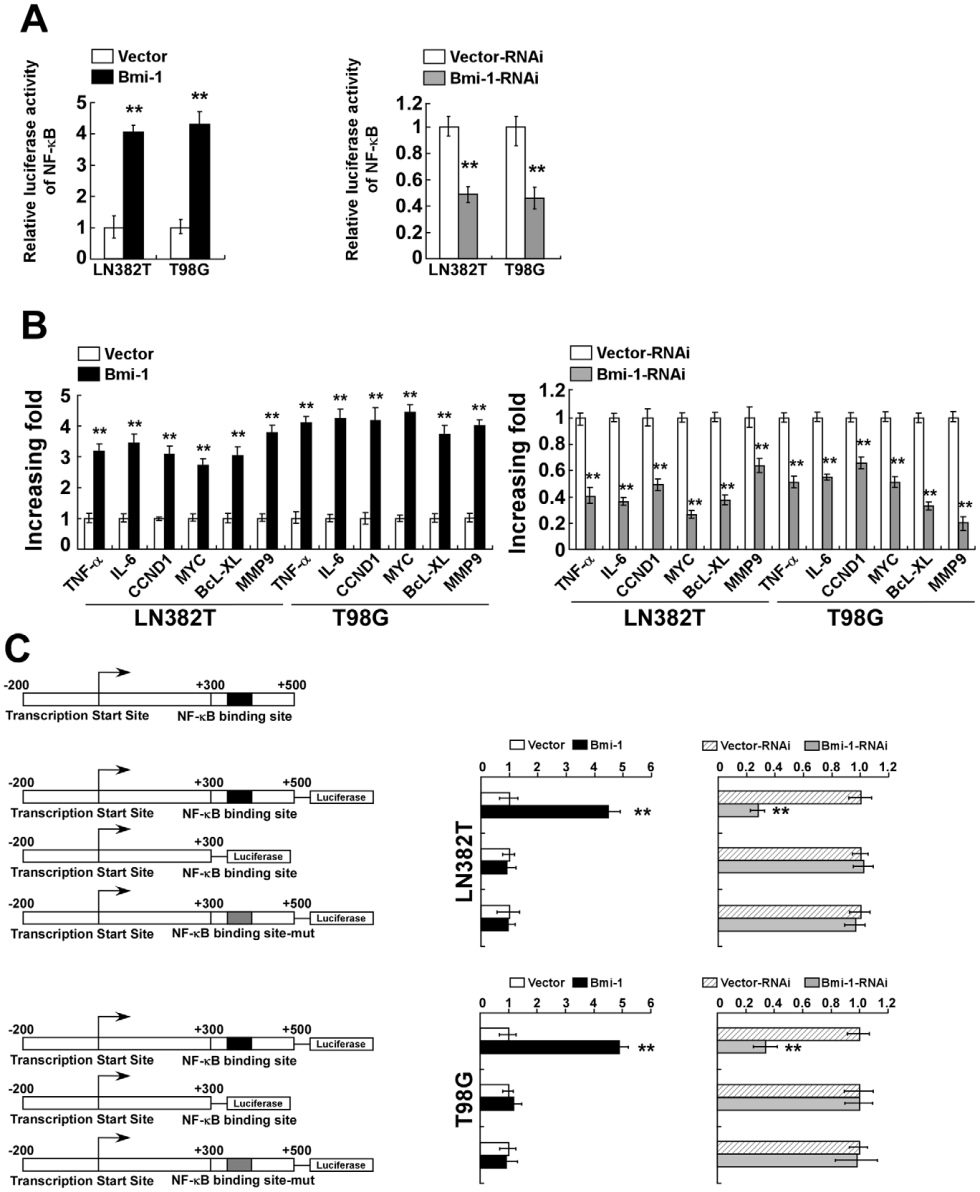
Bmi-1 induces NF-κB transcriptional activity. (A) Luciferase reporter assay of NF-κB transcriptional activity in Bmi-1 overexpressing (Bmi-1) and Bmi-1 silenced glioma cells (Bmi-1-RNAi), compared to the vector control cells respectively. (B) Real-time PCR analysis of NF-κB-regulated gene expression in vector-control, Bmi-1 overexpressing and Bmi-1-silenced glioma cells; GAPDH was used as the control gene. (C) Left, schematic illustration of luciferase reporter gene construction using cloned fragments of a human *VEGF-C* promoter. Right, transactivation activity of luciferase reporter genes driven by *VEGF-C* promoter fragments (as indicated on the left) in vector-control, Bmi-1 overexpressing and Bmi-1 silenced glioma cells. Luciferase activity was normalized to *Renilla* luciferase activity. Vector: pMSCV-vector, Vector-RNAi: pSuper-retro-puro-vector. Error bars represent the mean ± SD of three independent experiments; ** *P*<0.01.

We have previously shown that Bmi-1 induces NF-κB activation in glioma cells [22]. To determine whether NF-κB signaling mediates Bmi-1-promoted glioma angiogenesis, we first examined whether Bmi-1 expression enhances the transcriptional activity of NF-κB in LN382T and T98G glioma cells. As shown in Figure 5A, Bmi-1 overexpression induced transcription activity of NF-κB whereas siRNA knockdown of Bmi-1 reduced NF-κB activities. Furthermoe, the levels of several NF-κB target genes, *TNF-α*, *IL-6*, *CCND1*, *MYC*, *BcL-XL* and *MMP-9*, were upregulated in Bmi-1-overexpressing, but downregulated in Bmi-1-silenced glioma cells (Figure 5B). Additionally, compared to that in control cells, the activity of a *VEGF-C* promoter that contains a NF-κB binding site was significantly increased in Bmi-1-overexpressing but markedly decreased in Bmi-1-silenced glioma cells. Either a *VEGF-C* promoter fragment lacking the NF-κB binding site or mutation of the NF-κB binding site in the *VEGF-C* promoter region showed no NF-κB activity in either Bmi-1-overexpressing and -silenced glioma cells (Figure 5C). Taken together, these results suggest that Bmi-1 expression modulates the transactivation activity of the *VEGF-C* promoter harboring the NF-κB binding site in glioma cells.

**Figure 6:**
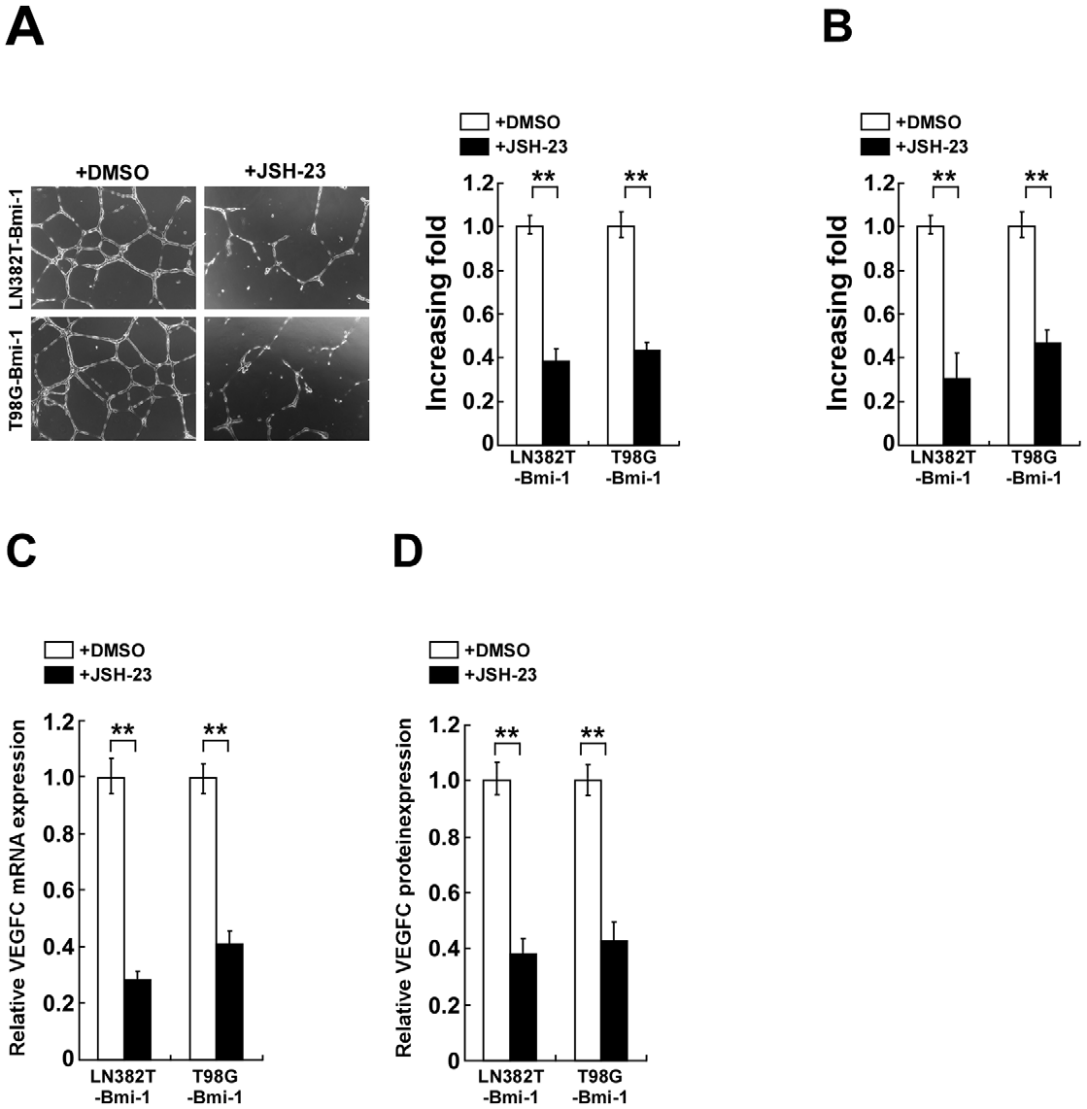
Bmi-1 promotes an angiogenesis phenotype in glioma cells via activation of the NF-κB. Bmi-1-overexpressing glioma cells were treated with a NF-κB inhibitor, JSH-23. (A) Representative images (left) and quantification (right) of HUVEC formed tube-like structures on Matrigel-coated plates with CM derived from indicated cultured cells. (B) Quantification of migrated HUVEC cells treated with indicated conditioned medium analyzed in a Transwell migration assay. (C) Real-time PCR quantification of *VEGF-C* mRNA expression levels in DMSO (control) and JSH-23 treated cells. Levels of mRNA expression are presented as fold increase relative to vector-control cells and normalized to GAPDH. (D) ELISA for VEGF-C protein expression in cell supernatants. Error bars represent the mean ± SD of three independent experiments; ** *P*<0.01.

Lastly, we examined whether Bmi-1 induced angiogenesis of glioma cells via activating NF-κB signaling. As shown in Figure 6 A *and* B, CM derived from Bmi-1 overexpressing glioma cells stimulated tube-like structures formed by HUVEC cells and HUVEC cell migration. Conversely, Bmi-1 stimulation of *in vitro* HUVEC angiogenic activities was markedly impeded by a specific NF-κB inhibitor JSH-23 that attenuates the transcriptional activity of NF-κB [31]. Furthermore, JSH-23 treatment also attenuated Bmi-1 induction of VEGF-C expression in glioma cells (Figure 6 C and D). Additionally, the HUVEC tube formation and migratory ability induced by Bmi-1 could be significantly reduced by treatment with a VEGF-C inhibitor (VEGFR3-Fc) (Figure S1 A and B). Collectively, the data suggest that functional NF-κB activation and the consequent VEGF-C expression are essential for the development of the pro-angiogenic phenotype of glioma cells induced by Bmi-1.

## Discussion

The major finding of this study is that upregulated Bmi-1 promotes angiogenesis in human gliomas *in vitro* and *in vivo*. Our results also suggest that the pro-angiogenic activity of Bmi-1 overexpression is associated with an activation of NF-κB signaling and the consequent upregulation of VEGF-C.

Previous studies have reported that knocking down of endogenous Bmi-1 diminishes secretion of VEGF from lung adenocarcinoma cells *in vitro*, leading to promoted angiogenesis in lung adenocarcinoma [32]. Moreover, co-expression of Bmi-1 and RasV12 promotes hepatocellular carcinoma (HCC) formation in mice, and the induced muring tumors resemble human HCC. Additionally, Bmi-1 promotes hepatic angiogenesis *in vivo* through cooperating with other oncogenic genes [33]. In this study, we demonstrate for the first time that Bmi-1 also promotes tumor angiogenesis in malignant gliomas. Our findings that Bmi-1 modulates the NF-κB/VEGF-C signaling pathway to mediate an angiogenesis phenotype in human gliomas further suggests that Bmi-1 may represent a prospective therapeutic target for anti-angiogenic strategies against the disease.

It is established that poor survival of patients with glioma can be largely attributed to the highly invasive nature of tumor, and the invasive progression is closely attributed to tumor angiogenesis [2]. Moreover, both invasion and angiogenesis result in destruction of surrounding brain tissue, and these characteristics of glioma cells correlates with patients prognosis [34]–[37]. While many studies have been conducted to investigate the oncogenesis and progression of glioma, the molecular mechanisms involved in regulating glioma angiogenesis remain incompletely understood. VEGF-C, a member of the VEGF family, plays an important role in angiogenesis, lymphangiogenesis and endothelial cell growth and survival. VEGF-C has been found to be upregulated in gliomas and associated with the progression and prognosis of the disease [38]. Of note, VEGF-C is also a direct target regulation of NF-κB signaling, and VEGF-C stimulates neovascularization through binding and activating VEGFR-2- and VEGFR-3-mediated cellular signaling [39]. Thus, our finding that Bmi-1 upregulates VEGF-C expression in glioma cells through NF-κB activation further underscores the importance of VEGF-C in glioma angiogenesis and provides a basis for using VEGF-C as a surveillance biomarker during anti-Bmi-1 therapy.

On the other hand, MMP-9 and IL-8, both of which are downstream target genes of NF-κB, have also been found to be upregulated in glioma cells [40], [41]. Furthermore, MMP-9 and IL-8 expression are further increased in Bmi-1-overexpressing glioma cells (Figure 5, and Figure S2). It has been reported that expression of MMP-9 closely correlates with tumor angiogenesis [42]. MMP-9 has also been shown to trigger an angiogenic switch during tumor progression by releasing matrix-bound VEGF, making the latter available for interaction with VEGF receptors [43]. In addition, IL-8 is expressed and secreted at high levels in human gliomas and is critical to gliomas neovascularity and progression [44]. Inducing IL-8 expression in glioma cells could promote glioma growth and angiogenesis through NF-κB signaling or other inflammatory stimuli [41]. Although the mechanism via which MMP-9 and IL-8 induce inflammatory stimuli glioma angiogenesis remains to be better understood, it would be of great interest to further evaluate whether and how in gliomas they cooperates with VEGF-C to promote neovascularization, due to the fact that these key angiogenic factors are all subject to the modulation of one upstream regulator, Bmi-1.

In conclusion, we demonstrate in this study that Bmi-1 induces glioma angiogenesis both *in vitro* and *in vivo*, and upregulation of Bmi-1 leads to increased VEGF-C expression through activating NF-κB signaling. Conversely, inhibition of NF-κB activity markedly reduces Bmi-1 stimulated angiogenic activities and attenuates Bmi-1 upregulation of VEGF-C. Our data provides new insights to the development of novel strategies to inhibit tumor angiogenesis in glioma by targeting Bmi-1, suggesting a necessity of further investigations of the anti-glioma therapeutic value of Bmi-1.

## Supporting Information

Figure S1 **VEGFC promotes glioma cell-induced** ***in vitro*** **angiogenesis.** Bmi-1-overexpressing glioma cells were treated with a VEGFC inhibitor, VEGFR3-Fc. (A) Representative images (left) and quantification (right) of HUVEC tubule formation on Matrigel-coated plates with CM derived from indicated cells. (B) Quantification of migrated HUVEC cells treated with indicated CM analyzed in a Transwell migration assay. ** *P*<0.01.
(TIF)

Figure S2 **Real-time PCR quantification of** ***IL-8*** **mRNA expression in vector-control cells (Vector) and Bmi-1-overexpressing cells (Bmi-1) (left), RNAi-control cells (vector-RNAi) and Bmi-1-downregulated cells (Bmi-1-RNAi) (right).** (Primer information, *IL-8*- forward: TGCCAAGGAGTGCTAAAG; *IL-8*- reverse: CTCCACAACCCTCTGCAC). Levels of mRNA expression are presented as the fold increase relative to that in control cells and normalized to GAPDH. ** *P*<0.01.
(TIF)
