# Supplementary figures and images for: Bmi-1 Promotes Glioma Angiogenesis by Activating NF-κB Signaling
Source: PLoS One. 2013 Jan 31;8(1):e55527. doi: 10.1371/journal.pone.0055527 (PMC3561301; doi:10.1371/journal.pone.0055527)

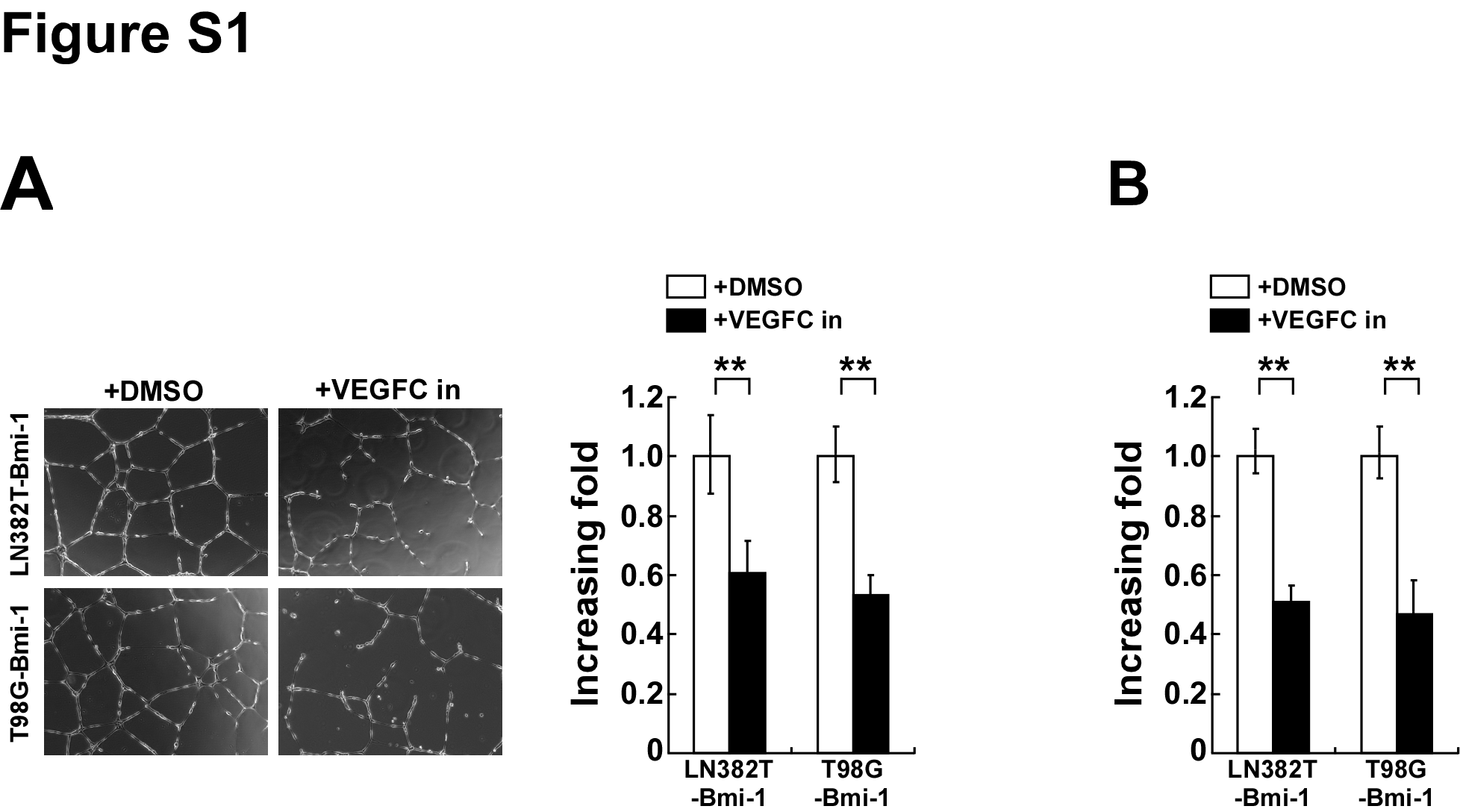

Supplement: Figure S1 — VEGFC promotes glioma cell-induced in vitro angiogenesis. Bmi-1-overexpressing glioma cells were treated with a VEGFC inhibitor, VEGFR3-Fc. (A) Representative images (left) and quantification (right) of HUVEC tubule formation on Matrigel-coated plates with CM derived from indicated cells. (B) Quantification of migrated HUVEC cells treated with indicated CM analyzed in a Transwell migration assay. ** P<0.01. (TIF) [file pone.0055527.s001.tif]

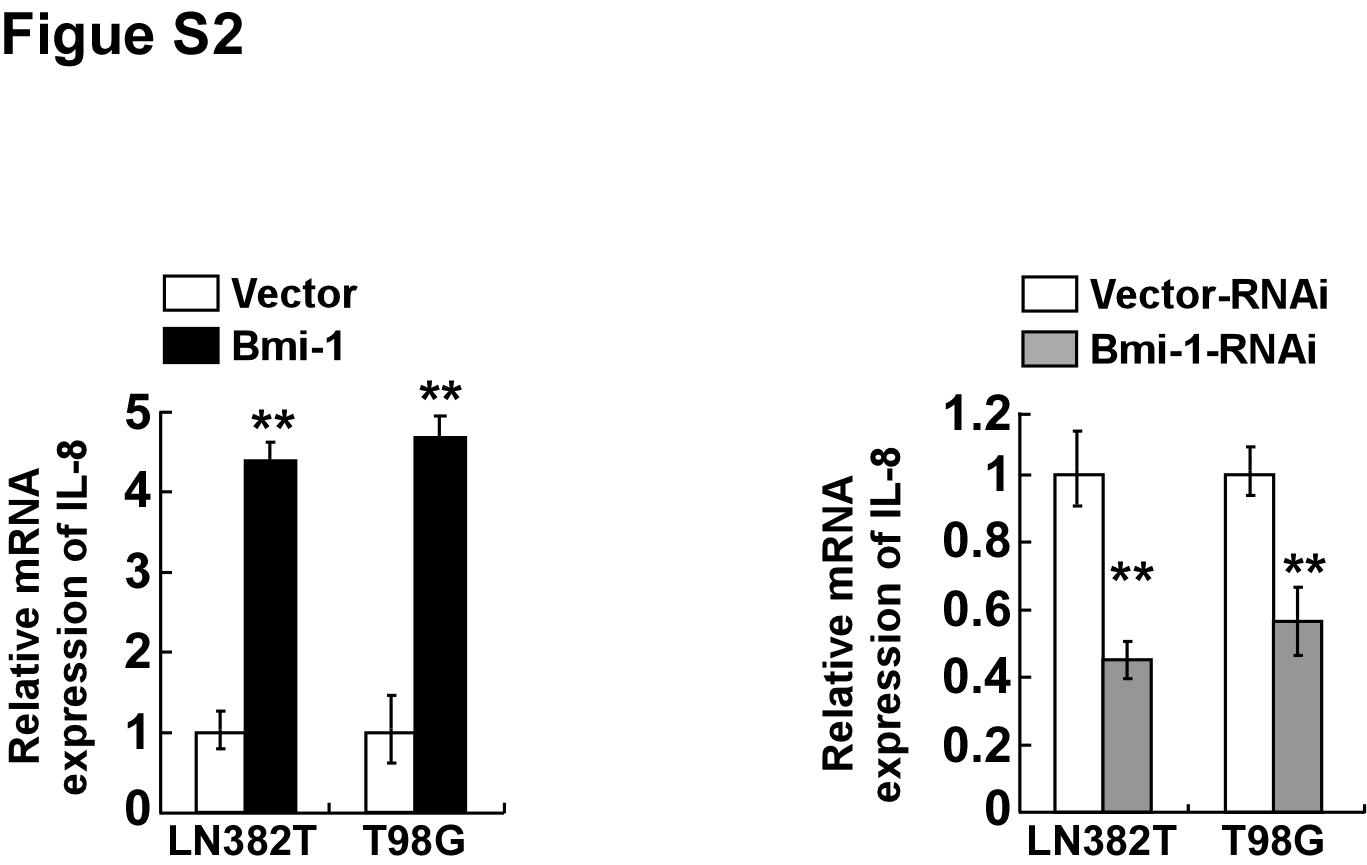

Supplement: Figure S2 — Real-time PCR quantification of IL-8 mRNA expression in vector-control cells (Vector) and Bmi-1-overexpressing cells (Bmi-1) (left), RNAi-control cells (vector-RNAi) and Bmi-1-downregulated cells (Bmi-1-RNAi) (right). (Primer information, IL-8- forward: TGCCAAGGAGTGCTAAAG; IL-8- reverse: CTCCACAACCCTCTGCAC). Levels of mRNA expression are presented as the fold increase relative to that in control cells and normalized to GAPDH. ** P<0.01. (TIF) [file pone.0055527.s002.tif]
